# Effectiveness of Mindfulness-Based Stress Reduction Therapy in Improving Quality of Life Among Anesthesiology Residents

**DOI:** 10.62641/aep.v54i2.1979

**Published:** 2026-04-15

**Authors:** Edith Casillas-Alvarez, Angélica Ortega-Barreiro, Alejandro González-Ojeda, Xóchitl Monteon Aspeitia, Jazmín Montserrat Guzmán Díaz, Carlos Enrique Capetillo-Texson, Guadalupe Castillo-Cardiel, Gabino Cervantes Guevara, Enrique Cervantes Pérez, Sol Ramirez Ochoa, Francisco Barbosa-Camacho, Clotilde Fuentes-Orozco

**Affiliations:** ^1^Departamento de Anestesiología, Unidad de Médica de Alta Especialidad (UMAE), Centro Médico Nacional de Occidente, Instituto Mexicano del Seguro Social (IMSS), 44329 Guadalajara, Mexico; ^2^Facultad de Medicina, Universidad de Colima, 28040 Colima, Mexico; ^3^Unidad de Investigación Biomédica 02, Unidad Médica de Alta Especialidad (UMAE), Centro Médico Nacional de Occidente, Instituto Mexicano del Seguro Social (IMSS), 44329 Guadalajara, Mexico; ^4^Departamento de Bienestar y Desarrollo Sustentable, Centro Universitario del Norte, Universidad de Guadalajara, 46200 Colotlán, Mexico; ^5^Departamento de Medicina Interna, Hospital Civil de Guadalajara Fray Antonio Alcalde, 44280 Guadalajara, Mexico; ^6^Departamento de Psiquiatría, Hospital Civil de Guadalajara Fray Antonio Alcalde, 44280 Guadalajara, Mexico

**Keywords:** mindfulness-based stress reduction, quality of life, medical residency, anesthesiology

## Abstract

**Background:**

Anesthesiology residents face high levels of occupational stress that can negatively impact their well-being and quality of life. Mindfulness-Based Stress Reduction (MBSR) has emerged as a promising intervention to support mental health in healthcare professionals. To evaluate the effectiveness of a modified MBSR program on quality of life among anesthesiology residents.

**Material and Methods:**

This prospective, single-group pre-post intervention study included 27 third-year anesthesiology residents (mean age 28.8 ± 1.9 years; 59.3% female) from a tertiary care hospital. Participants completed a 6-week modified MBSR program consisting of three 15-minute sessions per week. Quality of life was assessed using the Short Form-36 (SF-36) Health Survey before and after the intervention. Pre-post comparisons were conducted using paired *t*-tests and Wilcoxon signed-rank tests as appropriate.

**Results:**

Significant improvements were observed in five of eight SF-36 domains: Physical Functioning (91.67 ± 11.43 vs. 97.96 ± 4.65,* p =* 0.001), Role-Physical (60.19 ± 33.44 vs. 81.48 ± 22.56,* p =* 0.006), Bodily Pain (74.81 ± 19.01 vs. 89.44 ± 15.60,* p =* 0.049), Vitality (48.33 ± 15.75 vs. 71.67 ± 17.26,* p =* 0.001), and Mental Health (59.89 ± 13.39 vs. 78.96 ± 14.73,* p =* 0.004). No significant sex-based differences in treatment response were observed.

**Conclusions:**

A brief, modified MBSR program significantly improved multiple dimensions of quality of life in anesthesiology residents. These findings support the integration of mindfulness-based interventions into residency training programs to enhance resident well-being.

## Introduction

Healthcare workers (HCWs) face continuous exposure to stressors, including long working hours, night shifts, and witnessing patient suffering [1]. These factors contribute to the progressive development of psychological and physical stress, leading to psychiatric disorders such as anxiety, depression, and burnout [2]. Mindfulness-based interventions have emerged as promising approaches to address these challenges. Mindfulness is a state of nonjudgmental awareness of one’s thoughts, feelings, bodily sensations, and the surrounding environment [3]. Mindfulness-Based Stress Reduction (MBSR) is an evidence-based, standardized 8-week group intervention developed by Kabat-Zinn [4], incorporating meditation, yoga, and body awareness practices.

Studies consistently report high levels of mental distress among medical residents across various specialties [5, 6, 7]. A study conducted in China with 994 physicians reported the following prevalence rates: high perceived stress (43%), depression (36%), anxiety (20%), burnout (43%), low quality of life (36%), and suicidal ideation (27%) [8]. Mindfulness-based interventions have proven effective in supporting mental and physical well-being. In addition, this enhance the healthcare system functionality by promoting professionalism, collaboration, and effective communication [9]. On a personal level, mindfulness training helps HCWs manage their emotions by improving their ability to identify, observe, and regulate emotional responses [10].

Neuroimaging research demonstrates that mindfulness practice leads to structural brain changes. Voxel-based morphometry studies have observed significant increases in gray matter volume (GMV), particularly in the right anterior insula, the left and right inferior temporal gyri, and the right hippocampus [11]. A systematic review and meta-analysis of mindfulness-related changes in gray matter identified the right anterior ventral insula as the most consistent structure showing GMV increases across studies. An additional functional connectivity analysis revealed significant correlations between this structure and the bilateral insulae, as well as the anterior cingulate and adjacent paracingulate gyri [12].

Medical education has increasingly incorporated mindfulness training in recent decades. Since 1985, the University of Massachusetts Medical School has included an MBSR program in its curriculum, emphasizing the importance of early implementation in healthcare education [13]. Building on this foundation, we selected anesthesiology residents for the present study due to the unique demands of their clinical environment. They experience high levels of acute stress, prolonged operating room hours, continuous exposure to patient risk and pain, and the need for rapid, high-stakes decision-making. These characteristics make them an ideal population for evaluating the effectiveness of mindfulness-based interventions such as MBSR, which has been shown to reduce subjective stress and anxiety [14].

The program aimed to increase participants’ awareness of their thoughts, emotions, and all stimuli entering their field of perception while helping them recognize their habitual responses to stress. In this sense, the intervention serves as a tool to counteract maladaptive thinking patterns, such as rumination, which is considered a key source of psychological stress and is linked to both depression and anxiety [15, 16]. Specifically, MBSR promotes discipline through regular mindfulness meditation, enhances focus by training attention on the present moment, and builds resilience by encouraging consistent effort and perseverance in the face of stress [17]. Based on these considerations, this study aimed to assess the effectiveness of an MBSR intervention in enhancing the quality of life of anesthesiology residents.

## Methods

### Study Design and Setting

A prospective, pre-post intervention study was conducted from July 1 to August 31, 2022, at the Hospital de Especialidades Centro Médico Nacional de Occidente (CMNO), Instituto Mexicano del Seguro Social (IMSS). The study population consisted of third-year anesthesiology residents at this institution. The study was approved by the Hospital de Especialidades Centro Médico Nacional de Occidente Ethics Committee 1301 (ethics approval number R-2021-1301-084) and followed the ethical principles and guidelines of the Declaration of Helsinki.

### Participant Selection

We included third-year anesthesiology residents aged 27 to 36 years who provided informed consent. Residents with confirmed clinical diagnoses of any physical or psychological illness, as well as those who refused to participate or were lost to follow-up, were excluded. However, residents who reported subjective discomfort or subjective functional impairment without a formal diagnosis were not excluded. All participants volunteered after receiving detailed explanations of the study’s nature and procedures. Data confidentiality was guaranteed, and participants were informed that the obtained information would be used exclusively for research purposes. Once participants verbally agreed to participate, they signed informed consent forms before initiating the assessments. None of the participants received economic compensation for their participation.

Given that there were 28 third-year anesthesiology residents based at Centro Médico Nacional de Occidente, all eligible residents were invited to participate. Twenty-seven residents completed the study, yielding a 96.4% response rate. This study was not registered in a clinical trial database as it involved a non-randomized educational intervention without clinical outcomes.

### SF-36 Questionnaire

Quality of life was assessed using the Short Form-36 Health Survey (SF-36), a validated instrument developed by Ware and Sherbourne [18]. To evaluate health-related quality of life across eight domains: physical functioning, role limitations due to physical health (role-physical), bodily pain, general health, vitality, social functioning, role limitations due to emotional problems (role-emotional), and mental health. Each domain score ranges from 0 to 100, with higher scores indicating better perceived health status. The SF-36 has demonstrated acceptable internal consistency across populations, with a reported global Cronbach’s alpha coefficient of α = 0.87 in previous studies. The specific items for each domain are as follows:

• Physical Functioning: Items 3, 4, 5, 6, 7, 8, 9, 10, 11, and 12

• Role-Physical: Items 13, 14, 15, and 16

• Bodily Pain: Items 21 and 22

• General health: Items 1, 33, 34, 35, and 36

• Vitality: Items 23, 27, 29, and 31

• Social function: Items 20 and 32

• Role Emotional : Items 17, 18, and 19

• Mental health: Items 24, 25, 26, 28, and 30

### Intervention

The intervention consisted of a modified MBSR program adapted from the original 8-week protocol developed by Kabat-Zinn [4]. Participants attended mindfulness sessions three times per week, with each session lasting approximately 15 minutes, for a total duration of 6 weeks. This modified version differs from the original MBSR program in terms of session frequency (three times per week versus weekly) and session duration (15 minutes versus 2–2.5 hours). These modifications were necessary to accommodate the demanding workload inherent to anesthesiology residency training and to ensure active participation of the study subjects. The themes, content, and techniques of each session are summarized in Table 1.

**Table 1.** **Weekly content of the modified MBSR intervention**.

Week	Developed topic	Content summary	Main techniques used
1	Breathing	Breathing is used as an anchor to enhance focus, calm the mind, and foster present-moment awareness.	Anchoring in the present, conscious observation, emotional regulation
2	Body awareness and sound acceptance	Body scan to observe sensations and cultivate acceptance of external sounds without reaction.	Sensory exploration, non-reactivity, sound awareness
3	Thought distancing and present experience	Recognizing thoughts as transient events and anchoring attention to physical and emotional sensations.	Non-judgmental observation, mindfulness, gratitude practice
4	Pain and emotional regulation	Observing physical discomfort and painful emotions without avoidance, enhancing resilience.	Body scanning, emotional observation, relaxation, acceptance
5	Gratitude and loving-kindness	Fostering appreciation of positive experiences and practicing compassion for self and others.	Attention to positive events, metta meditation, connection
6	Open consciousness	Cultivating non-judgmental awareness of all arising experiences without clinging or avoidance.	Open monitoring, broad awareness, integration with environment

MBSR, Mindfulness-Based Stress Reduction.

### Study Procedure

Following participant recruitment and registration, researchers administered a baseline assessment using the SF-36 questionnaire before the intervention began. Participants then completed the 6-week modified MBSR program as described above. Within one week after the final session, the SF-36 questionnaire was re-administered to assess post-intervention outcomes.

### Statistical Analysis

Descriptive statistics were used to summarize participant characteristics and outcome measures. Qualitative variables were presented as frequencies and percentages, while quantitative variables were expressed as means and standard deviations. The Kolmogorov-Smirnov test was used to assess normality of distribution, which confirmed normal distribution of the data. For inferential analyses, paired Student’s *t*-test was used to compare pre- and post-intervention scores for normally distributed variables, while the Wilcoxon signed-rank test was applied as appropriate. A *p*-value of less than 0.05 was considered statistically significant. All statistical analyses were performed using SPSS version 26.0 (IBM Corp., Armonk, NY, USA).

## Results

Twenty-seven third-year anesthesiology residents who met the inclusion criteria participated in this study. The mean age was 28.8 ± 1.9 years (range 27–36 years). Of the participants, 11 (40.7%) were male and 16 (59.3%) were female. All participants completed both pre- and post-intervention assessments.

Analysis of pre- and post-intervention SF-36 scores revealed statistically significant improvements in five of the eight assessed domains (Table 2). Significant increases were observed in Physical Functioning (*p* = 0.001), Role-Physical (*p* = 0.006), Bodily Pain (*p* = 0.049), Vitality (*p* = 0.001), and Mental Health (*p* = 0.004). Although improvements were noted in General Health (*p* = 0.157), Role-Emotional (*p* = 0.079), and Social Functioning (*p* = 0.058), these changes did not reach statistical significance.

**Table 2.** **Comparison of SF-36 domain scores before and after MBSR intervention (n = 27)**.

Domain	Pre-MBSR Mean ± SD	Post-MBSR Mean ± SD	t	*p*-value
Physical Functioning	91.67 ± 11.43	97.96 ± 4.65	–3.573	0.001
Role-Physical	60.19 ± 33.44	81.48 ± 22.56	–3.793	0.006
Bodily Pain	74.81 ± 19.01	89.44 ± 15.60	–3.911	0.049
General Health	68.37 ± 18.02	79.81 ± 15.47	–2.942	0.157
Vitality	48.33 ± 15.75	71.67 ± 17.26	–8.153	0.001
Social Function	66.77 ± 23.24	85.18 ± 18.68	–4.013	0.058
Role-Emotional	39.24 ± 27.55	66.63 ± 30.67	–4.254	0.079
Mental Health	59.89 ± 13.39	78.96 ± 14.73	–7.263	0.004

Abbreviations: MBSR, Mindfulness-Based Stress Reduction; SD, standard deviation; SF-36, Short Form-36.

To explore potential sex differences in treatment response, we conducted separate analyses for male (n = 11, Table 3) and female (n = 16, Table 4) participants. Pre- and post-intervention scores were compared using the Wilcoxon signed-rank test for non-parametric variables and paired *t*-test for parametric variables, according to their distribution. Both male and female subgroups showed statistically significant improvements in most SF-36 domains. To formally test whether sex influenced the magnitude of treatment response, we performed a 2 × 2 repeated measures Analysis of Variance (ANOVA) (time × sex interaction). This analysis revealed no statistically significant sex-based differences in any of the eight SF-36 domains (Table 5), indicating that the intervention was equally effective across both sexes.

**Table 3.** **Pre and post intervention differences in the male group**.

	Male (Pre/Post)	Stat	*p* value
Physical Functioning	89.09 ± 13.93/97.72 ± 5.17	21*	0.027
Role-Physical	59.09 ± 34.04/86.36 ± 20.50	36*	0.010
Bodily Pain	74.81 ± 19.01/91.36 ± 17.01	3.76**	0.009
General Health	64.54 ± 18.07/80.45 ± 17.38	3.56**	0.013
Vitality	49.09 ± 13.38/73.18 ± 20.76	4.91**	0.003
Social Function	62.77 ± 24.38/85.22 ± 22.23	4.49**	0.005
Role-Emotional	39.38 ± 29.11/54.52 ± 34.23	1.45**	0.177
Mental Health	57.81 ± 13.78/78.54 ± 17.09	4.14*	0.005

*Wilcoxon matched pairs test, **Paired *t*-test.

**Table 4.** **Pre and post intervention differences in the female group**.

	Female (Pre/Post)	Stat	*p* value
Physical Functioning	93.43 ± 9.43/98.12 ± 4.42	28*	0.017
Role-Physical	60.94 ± 34.11/78.13 ± 23.94	2.03**	0.060
Bodily Pain	71.01 ± 18.07/88.12 ± 14.98	69*	0.018
General Health	71.01 ± 18.07/79.38 ± 14.59	1.44**	0.169
Vitality	47.81 ± 17.60/70.62 ± 15.04	6.34**	0.001
Social Function	69.53 ± 22.80/85.25 ± 16.59	2.23**	0.037
Role-Emotional	39.15 ± 27.40/74.96 ± 25.83	4.62**	0.002
Mental Health	61.31 ± 13.38/79.25 ± 13.48	6.14**	0.001

*Wilcoxon matched pairs test, **Paired *t*-test.

**Table 5.** **Comparison between men and women before and after MBSR intervention of the categories of the SF-36 questionnaire**.

	F*	*p* value
Physical Functioning	102	0.279321
Role-Physical	109.5	0.387923
Bodily Pain	111	0.602118
General Health	0.95209	0.350166
Vitality	0.215419	0.831189
Social Function	0.724799	0.475305
Role-Emotional	–1.6276	0.116149
Mental Health	0.514511	0.611414

*RM-ANOVA. MBSR, Mindfulness-Based Stress Reduction.

## Discussion

This study evaluated the impact of a modified MBSR program on quality of life among anesthesiology residents. Following six weeks of brief, structured mindfulness sessions, participants demonstrated significant improvements in five of eight SF-36 domains: Physical Functioning, Role-Physical, Bodily Pain, Vitality, and Mental Health. These findings suggest that even abbreviated mindfulness-based interventions can yield meaningful benefits for medical residents working in high-stress clinical environments, and importantly, these benefits appear to be consistent across both male and female participants.

Our results align with previous studies demonstrating the feasibility and effectiveness of MBSR in residency training programs. Lebares *et al*. [19, 20] reported improvements in general well-being and productivity among surgical residents following an eight-week MBSR program, findings consistent with the vitality improvements observed in our study. Notably, our six-week modified program achieved comparable benefits, suggesting that MBSR can be effectively adapted to accommodate the demanding schedules of medical trainees without substantially compromising outcomes. Similarly, studies in other specialties, including otolaryngology [21, 22, 23], pediatrics [24], and primary care [25], have documented reductions in stress and burnout alongside improvements in emotional regulation and focus. These consistent findings across diverse residency programs support the broader applicability of mindfulness-based interventions in graduate medical education.

The improvements in mental health and vitality observed in our study are particularly noteworthy given the critical role these domains play in maintaining cognitive performance and clinical decision-making in anesthesiology. Previous research has shown that mindfulness training enhances emotional resilience and stress management among medical students and residents [15, 26, 27], skills that are essential for managing the acute stressors inherent to anesthesiology practice. Furthermore, authors such as Minichiello *et al*. [28] and Szuster *et al*. [29] have advocated for the integration of MBSR into wellness curricula, citing benefits in personal development and interpersonal communication. Our findings provide empirical support for these recommendations and suggest that such programs may be especially valuable for residents in acute care specialties.

While our study did not include neuroimaging assessments, the observed improvements may be understood in the context of documented neurobiological changes associated with mindfulness practice. Neuroimaging research demonstrates that mindfulness training induces structural brain changes, including increased gray matter volume in regions such as the anterior insula, hippocampus, and prefrontal cortex, areas critical for emotional regulation, attention, and stress response [11, 12]. Additionally, functional connectivity changes in neural circuits subserving interoception and emotion processing have been documented following MBSR interventions. These neuroplastic changes provide a plausible mechanistic framework for understanding the improvements in vitality, mental health, and emotional functioning observed in our participants. Future studies incorporating neuroimaging assessments could help elucidate whether similar structural or functional brain changes occur with abbreviated MBSR protocols.

Our exploratory sex-stratified analysis revealed that both male and female participants experienced significant improvements across multiple SF-36 domains. Importantly, repeated measures ANOVA demonstrated no significant time × sex interaction effects, indicating that the intervention was equally effective for both sexes. This finding is consistent with previous literature suggesting that mindfulness-based interventions produce comparable benefits across diverse populations. These findings have important implications for residency training programs, particularly in high-acuity specialties such as anesthesiology. The demonstrated effectiveness of a brief, modified MBSR protocol suggests that meaningful improvements in resident well-being can be achieved even within the time constraints of clinical training. The consistent benefits observed across both male and female participants further support the broad applicability of such interventions. Given the growing mental health challenges faced by healthcare professionals and the documented high rates of burnout, stress, and depression among medical residents [5, 6, 7, 8], incorporating mindfulness-based programs into residency curricula may represent a practical and evidence-based approach to promoting resilience and enhancing quality of life. Our findings suggest that program directors need not implement the full eight-week traditional MBSR protocol to achieve clinically meaningful benefits, potentially reducing barriers to implementation.

This study has several important limitations that should be considered when interpreting the results. First, the single-group, pre-post design without a control group limits our ability to establish causality and control for potential confounding factors such as time effects, regression to the mean, or expectancy effects. Second, the small sample size (n = 27) from a single institution limits statistical power and generalizability of findings to other settings and populations. Third, reliance on self-reported measures may introduce response bias, and the lack of objective outcomes such as physiological markers, behavioral assessments, or long-term follow-up data represents a significant gap. Fourth, although participants with clinically diagnosed psychological disorders were excluded, we cannot rule out the presence of undiagnosed subclinical conditions, particularly given the relatively low baseline scores observed in the Role-Emotional domain, which may reflect situational stress common during residency training. Finally, the modified nature of the intervention—with shorter sessions (15 minutes versus 2–2.5 hours) and briefer overall duration (6 weeks versus 8 weeks)—deviates substantially from the standardized MBSR protocol, making direct comparisons with existing literature challenging.

Despite these limitations, this study provides preliminary evidence that a brief, modified MBSR program can improve multiple dimensions of quality of life among anesthesiology residents. The observed benefits in physical functioning, bodily pain, vitality, and mental health are particularly relevant given the demanding nature of anesthesiology training. Future research should employ randomized controlled designs with adequate sample sizes, include active control groups, incorporate objective outcome measures, and assess long-term sustainability of benefits. Additionally, studies exploring optimal intervention parameters (session duration, frequency, and overall program length) and investigating neurobiological correlates of observed improvements would enhance our understanding of how mindfulness interventions can be most effectively implemented in medical training. Given the escalating mental health crisis among healthcare professionals, developing evidence-based, feasible interventions to support resident well-being remains a critical priority.

## Conclusions

This study demonstrates that a brief, modified MBSR program can significantly improve quality of life among anesthesiology residents in a demanding clinical training environment. Significant improvements were observed in physical functioning, role-physical, bodily pain, vitality, and mental health, with benefits evident across both male and female participants. These findings suggest that mindfulness-based interventions can be feasibly integrated into residency training programs and may represent a practical approach to supporting resident well-being in high-stress medical specialties.

## Availability of Data and Materials

The datasets generated and analyzed in this study are available from the corresponding author upon reasonable request.
